# Tick and Tick-Borne Diseases: New Problems Providing New Possible Solutions

**DOI:** 10.3390/pathogens12010120

**Published:** 2023-01-11

**Authors:** Olivier Andre Sparagano

**Affiliations:** Department of Infectious Diseases and Public Health, Jockey Club College of Veterinary Medicine and Life Sciences, City University of Hong Kong, Hong Kong SAR, China; olivier.sparagano@cityu.edu.hk

Ticks and tick-borne diseases are responsible for enormous losses in animal and human life, which do not seem to become better as new data show surprising connections. For instance, tick bites (from *Ixodes ricinus*) were linked to new red meat allergies in patients ([1] such patients with IgE against galactose-α-1,3-galactose (known as α-Gal) showed severe allergic reactions after consumption of red meat).

It is also not completely clear how pathogens from different infection sources work together but a recent paper on patients with high hospitalization rates showed that such patients also had a high prevalence of antibodies against *Borrelia*, a possible coincidence of disabling or confusing the patient’s immune system to allow other pathogens to proliferate more easily [2], or unclear synergies between infectious agents? This could be a similar scenario between some HIV patients showing the highest prevalence of tick-borne pathogens [3]. These multiple, unrelated and complex infections are putting pressure on medical practitioners and veterinarians to successfully treat them, while co-infections between tick-borne pathogens, possibly transmitted by the same tick species, are creating a public-health problem to tackle [4,5,6].

Furthermore, ticks on their own are developing resistance, making it more difficult to prevent their proliferation or treat their infestations. Tick resistance to traditional treatments is not uncommon [7,8], creating a conundrum for practitioners but also opening new opportunities using plant-derived products [9].

Tick-borne pathogens are found in many different host species, from wildlife animals [10] to animals in zoos [11], some carrying zoonotic pathogens, putting at risk the workers looking after such animals.

It is important to identify the key risk factors and practices that can prevent or influence tick development. First, we need to find a consensus on taxonomy keys, where there has not always been full agreement between national taxonomic books [12]. Second, understanding the risk factors and practices (Knowledge, Attitude and Practice) from animal owners would ensure clarity in risk factors to be dealt with as a priority [13,14,15].

With such a development and ticks colonizing new areas, it is clear than new tick-surveillance systems are needed to raise the alarm as soon as they colonize a new bio-ecosystem. Surveillance of ticks and tick-borne diseases has been well documented [16,17] and modelling can help in such an empiric approach [18].

Climate change, a buzzword used perhaps too often and not appropriately all the time, seems to impact tick adaptation [19,20,21].

Not all is lost, as further genome sequencing allows us to better understand how to develop new drugs or new tick vaccines, including a lot of research now on the tick gut microbiome to also evaluate the potential of indirectly knocking down ticks through their own microbiome.

The tick vaccine did not start well but a lot was learnt from the first *Boophilus-microplus* (now known as *Rhipicephalus (Boophilus) microplus*)-related vaccines and new possibilities are emerging, meaning a tick vaccine could be available in the next few years [22,23] and an understanding of the tick immune system and tick immunobiology [24] is allowing us to prepare counter attacks.

The host–pathogen relationship could also provide new treatments if we also add the tick saliva microbiome [25] and tick gut microbiome to the equation [26,27].

A One-Health approach has to be kept in mind to avoid developing new treatment, which could work against ticks but be detrimental to animals, humans and their environment [28,29], while other parameters, such as biotic factors, should also be considered in the complex fight against ticks and the pathogens they can carry and transmit [30,31,32].

In conclusion, and hopefully as a way forward, we need to develop consensus diagnostics and methodologies [33], sharing data, not only molecular diagnostics, standardization [34], informative reviews [35] and international collaborations [36].

More challenges and successes are on the way, which we, as a united scientific community, must be ready for.
